# Downregulation of CCL22 and mutated *NOTCH1* in tongue and mouth floor squamous cell carcinoma results in decreased Th2 cell recruitment and expression, predicting poor clinical outcome

**DOI:** 10.1186/s12885-021-08671-1

**Published:** 2021-08-15

**Authors:** Xuejie Li, Zheqi Liu, Wenkai Zhou, Xiaofang Liu, Wei Cao

**Affiliations:** 1 Department of Oral and Maxillofacial Surgery, The First Affiliated Hospital of Zhengzhou University, Zhengzhou, 450052 People’s Republic of China; 2 Department of Oral and Maxillofacial & Head and Neck, Oncology, Shanghai Ninth People’s Hospital, Shanghai Jiao Tong University School of Medicine, Shanghai, 200011 People’s Republic of China; 3 National Center for stomatology, National Clinical Research Center For Oral diseases, Shanghai Key Laboratory of Stomatology, Shanghai, 200011 People’s Republic of China; 4 Department of Neurology, The First Affiliated Hospital of Zhengzhou University, Zhengzhou, 450052 People’s Republic of China

**Keywords:** Tongue and mouth floor SCC, Th2 cells, CCL22, NOTCH1, TCGA

## Abstract

**Objective:**

Tongue and mouth floor squamous cell carcinoma (T/MF SCC) exhibits a high rate of local recurrence and cervical lymph node metastasis. The effect of the tumor microenvironment on T/MF SCC remains unclear.

**Materials and methods:**

Transcriptome and somatic mutation data of patients with T/MF SCC were obtained from HNSC projects of the Cancer Genome Atlas. Immune infiltration quantification in early- (clinical stage I–II) and advanced-stage (clinical stage III–IV) T/MF SCC was performed using single sample Gene Set Enrichment Analysis and MCPcounter. Differentially expressed gene data were filtered, and their function was assessed through Gene Ontology and Kyoto Encyclopedia of Genes and Genomes analyses. Kaplan–Meier survival curve analysis and Cox regression model were conducted to evaluate the survival of patients with the CCL22 signature. Maftools was used to present the overview of somatic mutations.

**Results:**

In T/MF SCC, T helper (Th)2 cell counts were significantly increased in patients with early-stage disease compared to those with advanced-stage disease. Expression of the Th2 cell-related chemokine, CCL22, was downregulated in patients with advanced-stage T/MF SCC. Univariate and multivariate Cox analyses revealed that CCL22 was a good prognostic factor in T/MF SCC. A nomogram based on the expression of CCL22 was constructed to serve as a prognostic indicator for T/MF SCC. *NOTCH1* mutations were found at a higher rate in patients with advanced-stage T/MF SCC than in those with early-stage T/MF SCC, resulting in the inhibition of the activation of the NOTCH1-Th2 cell differentiation pathway. The expression levels of CCL22, GATA-3, and IL4 were higher in patients with early-stage T/MF SCC than in those with advanced-stage T/MF SCC.

**Conclusion:**

In T/MF SCC, high expression of CCL22 may promote the recruitment of Th2 cells and help predict a better survival. Mutations in *NOTCH1* inhibit the differentiation of Th2 cells, facilitating tumor progression through a decrease in Th2 cell recruitment and differentiation.

**Supplementary Information:**

The online version contains supplementary material available at 10.1186/s12885-021-08671-1.

## Introduction

Oral squamous cell carcinoma (OSCC) is one of the most commonly reported malignant tumors in human beings, accounting for 2% of all tumors [1]. Tongue and mouth floor squamous cell carcinoma (T/MF SCC) is the most common type of OSCC documented. T/MF SCC exhibits a high potential for local recurrence and metastasis to cervical lymph nodes. Although substantial progress has been achieved in the treatment of T/MF SCC, the five-year survival rate of patients presenting with T/MF SCC has not shown improvement over the last 20 years [2]. Therefore, a better understanding of the mechanism of T/MF SCC progression is crucial to improve the prognosis of patients with T/MF SCC.

Accumulating evidence indicates that the interaction between tumor cells and the tumor microenvironment (TME) is of remarkable significance for the occurrence and development of tumors [3–7]. The abundance of tumor stromal cells, such as infiltrating regulatory T cells (Tregs) or myeloid suppressor cells, and cytokines may contribute to cancer progression and invasiveness and to the occurrence of immunosuppression in patients with tongue SCC [8]. The correlation between the development of T/MF SCC and other immune microenvironment components remains unclear.

T helper (Th)2 cells undergo differentiation from naïve CD4^+^ T cells and mediate humoral immunity. Few studies indicate that increased abundance of Th2 cells can be considered to predict poor survival in colorectal and esophageal cancer [9, 10], while others suggest that high proportions of Th2 cells and expression of IL4, which is secreted by Th2 cells, may help predict a good prognosis in B cell non-Hodgkin’s lymphoma (NHL) [11]. The role of Th2 cells in T/MF SCC has not been studied thus far.

Accordingly, in this study, the immune infiltration in T/MF SCC was analyzed and compared between patients with early- and advanced-stage disease, based on data derived from the Cancer Genome Atlas (TCGA). Based on this analysis, Th2 cells were found to be significantly enriched in patients with early-stage T/MF SCC. Expression of Th2 cell-related chemokines and the entire status of gene mutations were investigated to explain the enrichment of Th2 cells observed in our study.

## Materials and methods

### RNA sequencing data from TCGA

Gene expression data of count and FPKM type and the clinical information of patients with T/MF SCC (178 cases) were downloaded from HNSCC projects of TCGA database (https://genome-cancer.ucsc.edu/). Patients with complete follow-up data were included. Then, FPKM data were transformed into transcripts per million reads (TPM) for further analyses. This study meets the publication guidelines of TCGA. All data used in this study were obtained from TCGA, and hence, ethics approval and informed consent were not necessary.

### Acquisition of somatic mutation data

Somatic mutation data were obtained from TCGA database via the GDC data portal (https://portal.gdc.cancer.gov/). From the four subtypes of data files, we selected “mutect2” data. We used the Maftools package [12] to prepare the Mutation Annotation Format (MAF) of somatic variants. The visualization of the mutation analysis was conducted using Maftools.

### Analysis of immune infiltration

The analysis of the immune infiltration in T/MF SCC was conducted using MCPcounter, and the single sample Gene Set Enrichment Analysis (ssGSEA) method was performed using the GSVA package (http://www.bioconductor.org/packages/release/bioc/html/GSVA.html) in R. Based on the signature genes reported in the literature [13, 14], the relative enrichment score of all immune cells was calculated based on the gene expression profile (TPM type) deduced for each tumor sample. The T-test and Spearman’s correlation were used to evaluate the association between CCL22 expression and the abundance of immune cells.

### Differentially expressed gene (DEG) analysis

Cases were divided into early stage (clinical stage I–II) and advanced stage (clinical stage III–IV) according to the clinical information. Then, expression data (count type) were compared between the two groups to identify DEGs using the DESeq2 package in R [15]. |log2FoldChange| > 1.5 and adjusted *P*-value < 0.05 were set as threshold values for DEGs.

### Functional analysis of DEGs

Gene Ontology (GO) and Kyoto Encyclopedia of Genes and Genomes (KEGG) analyses were conducted using the enrichGO and enrichKEGG functions of the clusterProfiler R package [16]. Adjusted *P* values (false-discovery rate) less than 0.05 were considered to be statistically significant. The visualization of GO and KEGG results was conducted using clusterProfiler R and the GOplot package [17].

### Tumor tissue samples

Eight paraffin-embedded samples of tongue SCC were collected from patients at the Ninth People’s Hospital, Shanghai Jiao Tong University School of Medicine. All patients were informed about the study and provided written informed consent, and the process was approved by the Ethics Committee of the Ninth People’s Hospital, Shanghai Jiao Tong University School of Medicine. The clinical information of patients is shown in Table 1.

**Table 1:** Clinical features of patients with tongue SCC

Patients	Labeling index			Clinical stage	T stage	N stage	Vitalstatus
	CCL22	GATA3	IL4				
1	25	25	0	IV	4	3	1
2	25	50	25	IV	4	1	1
3	25	50	25	III	2	2	1
4	50	50	25	III	2	2	0
5	150	100	50	II	2	0	0
6	150	150	75	II	2	0	0
7	100	150	50	II	2	0	0
8	150	75	50	I	1	0	0

### Immunohistochemical staining and semi-quantitative analysis

Paraffin-embedded tissues were subjected to staining for immunohistochemical detection of CCL22, GATA-3, and IL4. The slides were dried at 60 °C, dewaxed with methanol, and rehydrated with alcohol. Then, the slides were immersed in 3% hydrogen peroxide and subjected to labeling with anti-CCL22 (Abcam, ab124768), anti-GATA-3 (Affinity Biosciences, AF6233), and anti-IL4 (Bioworld, BS3501) antibodies overnight. The labeling index was semi-quantitatively assessed as the intensity of staining (0, 1, 2, or 3) multiplied by the percentage of positively stained epithelial thickness (25, 50%, or 75%) using the IHC Profiler function in the Image J software.

### Statistical analysis

Statistical analysis was performed using R (4.0.2). Comparison of gene expression between patients with early- and advanced-stage T/MF SCC, as well as the relationship between clinicopathological features and CCL22 expression, were analyzed using the T-test. Clinicopathological characteristics associated with overall survival (OS) were analyzed using the Cox regression and Kaplan–Meier methods. Multivariate Cox analysis was conducted to assess the influence of CCL22 expression on survival along with other clinical features. A receiver operating characteristic curve was generated to test the performance of the multiCox model using R package survivalROC. A nomogram was constructed based on the results of the multivariate analysis using the rms package in R. DCA curves were generated to test the performance of the nomogram using the rmda package. All hypothetical tests were two-sided, and *P*-values < 0.05 were considered significant in all tests.

## Results

### Th2 cell numbers are higher in patients with early-stage T/MF SCC than in those with advanced-stage T/MF SCC

**Fig. 1:**
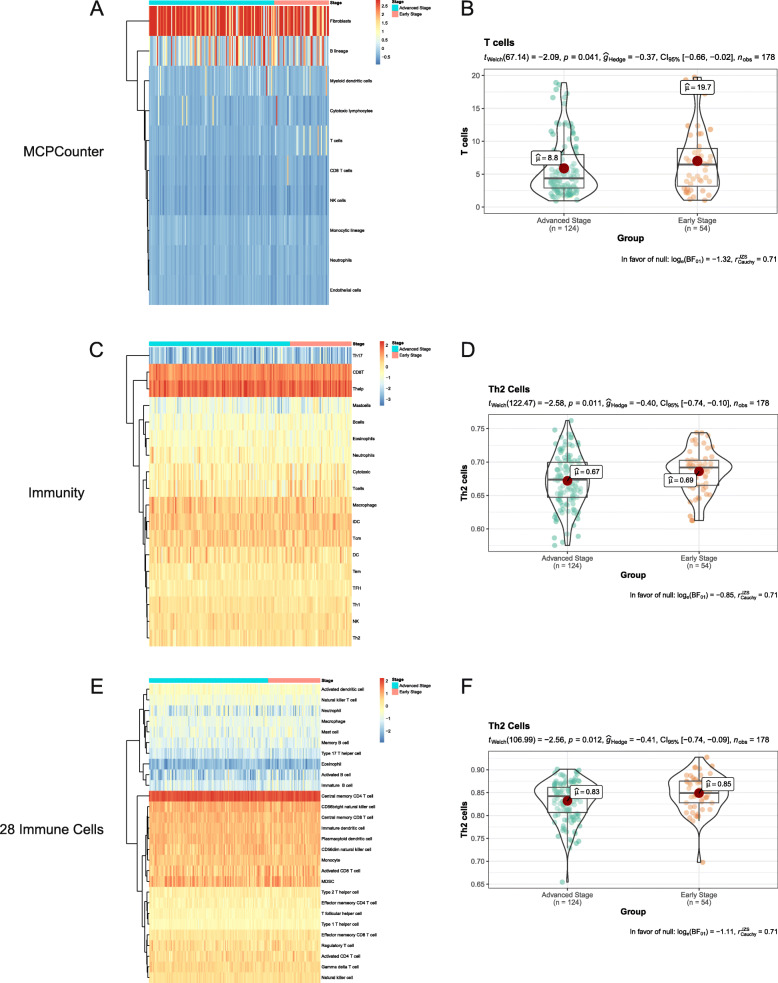
Data on infiltrating immune cells in samples derived from TCGA datasets. Th2 cell numbers were increased in early-stage T/MF SCC compared to advanced-stage T/MF SCC. (**A**) Distribution of tumor-infiltrating immune cells in TCGA samples calculated using MCPcounter. (**B**) Relative number of T cells in early- and advanced-stage T/MF SCC samples calculated using MCPcounter. (**C**) Distribution of tumor-infiltrating immune cells in TCGA samples calculated using ssGSEA and a gene signature from the literature [14]. This signature was termed as *Immunity*. (**D**) Relative numbers of Th2 cells in early- and advanced-stage T/MF SCC samples according to the *Immunity* signature. (**E**) Distribution of tumor-infiltrating immune cells in TCGA samples calculated using ssGSEA and information on a gene signature obtained from the literature [13]. This signature was termed as *28 Immune cells*. (**F**) Relative numbers of T cells in early- and advanced-stage T/MF SCC samples according to the *28 Immune cells* signature. In heatmaps, red represents a high distribution, while blue represents a low distribution

To compare the abundance of immune cells between early- and advanced-stage T/MF SCC samples, we first used the MCPcounter package to analyze the components of the TME in each sample (Fig. 1A). Among the immune cells, T cell numbers were significantly increased in patients with early-stage T/MF SCC compared to those with advanced-stage T/MF SCC (Fig. 1B, *P* = 0.041). To verify the type of T cells underwent changes in terms of frequency, ssGSEA was conducted using two types of gene signatures whose data were obtained from different studies [13, 14]. We defined these two signatures as *Immunity* [14] and *28 Immune Cells* [13], and they have been presented in Supplementary Information. The immune cell infiltration numbers are shown in Fig. 1C and E. Th2 cell numbers were significantly increased in patients with early-stage disease, compared to those with advanced-stage disease, in both gene signatures (Fig. 1D and F, *P* = 0.011 and *P* = 0.012 for *Immunity* and *28 Immune Cells*, respectively).

### Screening of DEGs between patients with early- and advanced-stage T/MF SCC, and functional cluster analysis

**Fig. 2:**
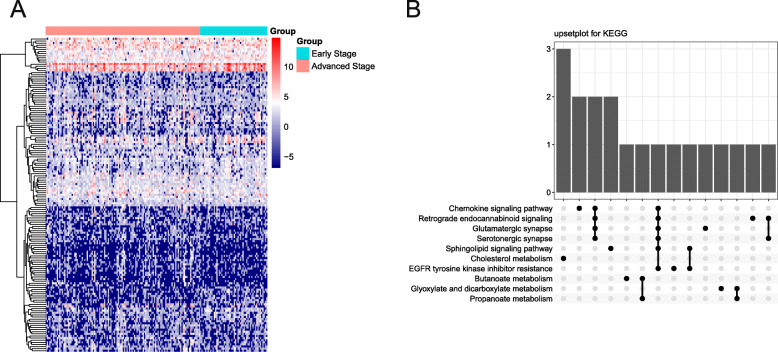
Screening of differentially expressed genes (DEGs) and functional analysis. (**A**) Heatmap illustrating DEGs between early- and advanced-stage T/MF SCC samples. (**B**) Upset plot for KEGG pathways. The bar chart on the top shows the number of genes contained in each group. The dotted lines at the bottom represent genes involved in each pathway

To explore the potential mechanism underlying the increase in Th2 cell count, we analyzed DEGs in early- and advanced-stage T/MF SCC samples. A total of 478 DEGs were identified, wherein the expression levels of 348 genes were upregulated and those of 130 genes were downregulated. Data on a total of 149 genes were filtered using the |log2FoldChange| > 1.5 and adjusted *P* < 0.05 thresholds (Fig. 2A). Among the genes, the expression levels of 43 were downregulated and those of 106 genes were upregulated. Then, the functions of DEGs in patients with T/MF SCC were predicted using GO and KEGG enrichment analyses. The top 10 GO enrichment items in the biological process, molecular function, and cellular component groups are shown in Fig. S1. KEGG analysis results showed that the chemokine signaling pathway was the first enriched item (Fig. 2B). Considering the increased number of Th2 cells in patients with early-stage T/MF SCC, we suggest that chemokines may influence Th2 cell aggregation in patients with this disease.

### CCL22 expression is upregulated in early-stage T/MF SCC and is positively correlated with the number of Th2 cells

**Fig. 3:**
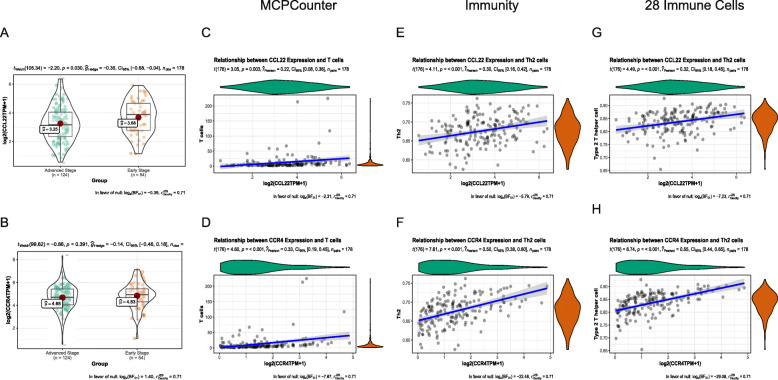
Correlation between CCL22/CCR4 expression and T/Th2 cell numbers. Expression of (**A**) CCL22 and (B) CCR4 in early- and advanced-stage T/MF SCC samples. Correlation between (**C**) CCL22 or (**D**) CCR4 expression and T cell numbers, according to MCPcounter. Correlation between (**E**) CCL22 or (**F**) CCR4 expression and Th2 cell numbers using the *Immunity* signature. Correlation between (**G**) CCL22 or (**H**) CCR4 expression and Th2 cell numbers using the *28 Immune Cells* signature. Violin plots presented on the margin of dotplots represent the distribution of CCL22/CCR4 expression and T/Th2 cell numbers

CCL22 and CCL17 are associated with the induction of chemotaxis in T cells, particularly Th2 cells, via binding to the chemokine receptor CCR4. We compared the expression of CCL22, CCL17, and CCR4 between early- and advanced-stage T/MF SCC samples and found that CCL22 and CCR4 expression levels were upregulated in the former (Fig. 3A and B), while no significant difference was noted for CCL17 expression (Fig. S2). Then, we analyzed the correlation between the expression of CCL22 or CCR4 and immune cell infiltration numbers using MCPcounter, *Immunity*, and *28 Immune Cells* groups using the Spearman’s correlation (Fig. 3C-H). The results indicated that both CCL22 and CCR4 expression levels were strongly and positively correlated with Th2 cell counts.

### Prognostic value of CCL22 in T/MF SCC

**Fig. 4:**
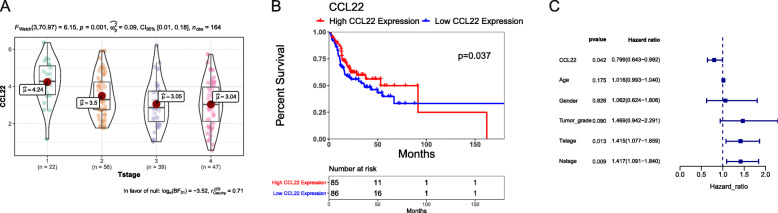
Clinical relevance and prognostic value of CCL22 expression. (**A**) CCL22 expression in T/MF SCC at different T stages. (**B**) Kaplan–Meier curve of overall survival according to high and low expression levels of CCL22. (**C**) Forest plot illustrating the independent prognostic factors

To further elucidate the mechanism by which CCL22 and CCR4 were involved in T/MF SCC development, we analyzed the correlation between the expression of CCL22 or CCR4 and clinical parameters. In this study, we analyzed the clinical information of 178 patients with T/MF SCC from TCGA database. The patients’ clinicopathological features are shown in Table 2. CCL22 expression was higher in the T1 and T2 stages than that in the T3 and T4 stages (Fig. 4A), while the expression of CCR4 did not correlate with the clinicopathological features. The expression of CCL22 in T/MF SCC tissues was classified as low or high according to its median value. A Kaplan–Meier OS curve was plotted to analyze the prognosis of patients with T/MF SCC expressing CCL22 at different levels. The log-rank test of OS revealed that high expression of CCL22 was significantly associated to a better prognosis (Fig. 4B, *P* = 0.037). Univariate and multivariate Cox regression analyses were performed to identify the independent prognostic factor in patients with T/MF SCC (Table 3 and Fig. S3). The results suggested that CCL22, but not CCR4, was an independent protective factor in patients with T/MF SCC (Fig. 4C). The AUC of the multivariate Cox model was 0.7683 (Fig. S4).

**Table 2:** Clinicopathological parameters of patients with tongue squamous cell carcinoma based on TCGA data

Characteristics	*n* = 178
Survival time (days, mean (SD))	798.92 (760.64)
Age (mean (SD))	59.19 (13.39)
Gender (%)	
Female	58 (32.6)
Male	120 (67.4)
Tumor grade (%)	
G1	21 (11.8)
G2	122 (68.5)
G3	35 (19.7)
T stage (%)	
T1	24 (14.0)
T2	56 (32.7)
T3	41 (24.0)
T4	50 (29.2)
N stage (%)	
N0	70 (42.7)
N1	24 (14.6)
N2	68 (41.5)
N3	2 (1.2)
Clinical stage (%)	
Stage I–II	54 (30.3)
Stage III–IV	124 (69.7)

**Table 3:** Univariate and multivariate Cox regression analyses of clinicopathological parameters and overall survival

Characteristics	Hazard ratio (95% CI) in univariate analysis	*P*-value in univariate analysis	Hazard ratio (95% CI) in multivariate analysis	*P*-value in multivariate analysis
CCL22	0.774 (0.641–0.934)	0.008	0.799 (0.643–0.992)	0.042
GATA-3	0.878 (0.701–1.100)	0.257		
CCR4	0.846 (0.674–1.063)	0.152		
Age	1.017 (0.998–1.036)	0.087	1.016 (0.993–1.040)	0.175
Gender	1.108 (0.693–1.771)	0.669	1.062 (0.624–1.806)	0.826
Tumor grade	1.415 (0.954–2.099)	0.084	1.469 (0.942–2.291)	0.090
T stage	1.528 (1.209–1.932)	0.000	1.415 (1.077–1.859)	0.013
N stage	1.518 (1.167–1.974)	0.002	1.417 (1.091–1.840)	0.009

### Establishment of survival prognostic models for T/MF SCC using CCL22

**Fig. 5:**
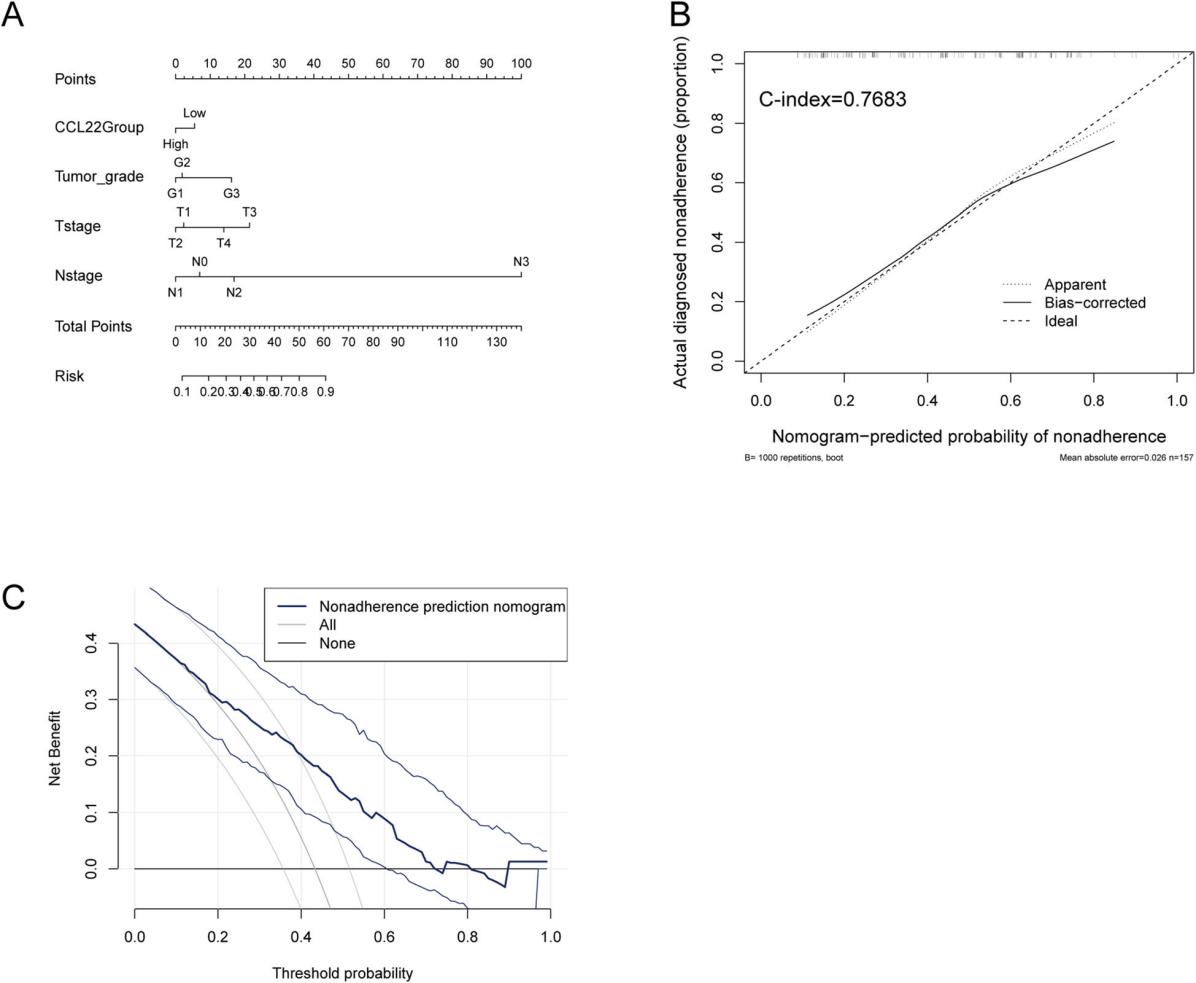
Establishment and evaluation of survival prognostic models for T/MF SCC. (**A**) Nomogram for predicting the probability of 1-, 3-, and 5-year survival of patients with T/MF SCC. (**B**) Calibration curve for evaluating the accuracy of the nomogram for patients with T/MF SCC. (**C**) Decision curve analysis for evaluating the performance of the nomogram. Lines on both sides represent confidence intervals

The previous results indicated that CCL22 was an independent prognostic factor in T/MF SCC; thus, we aimed to establish a predictive model for the OS by fitting data on CCL22 expression and other characteristics. A nomogram integrating data on CCL22 expression and other characteristics, including age, gender, tumor grade, T stage, and N stage, was constructed (Fig. 5A), wherein a worse prognosis was represented by a high score on the nomogram. The performance of the nomogram integrating data on CCL22 expression was evaluated using a calibration curve, and the C-index was estimated to be 0.7683 (Fig. 5B). We also performed a decision curve analysis to evaluate the performance of this prediction model. The prediction nomogram demonstrated a high benefit percentage (Fig. 5C). In summary, the nomogram integrating CCL22 expression may be considered a better model for predicting the survival of patients with T/MF SCC than individual prognostic factors.

### NOTCH1 mutation in advanced-stage T/MF SCC samples decreases Th2 cell differentiation

**Fig. 6:**
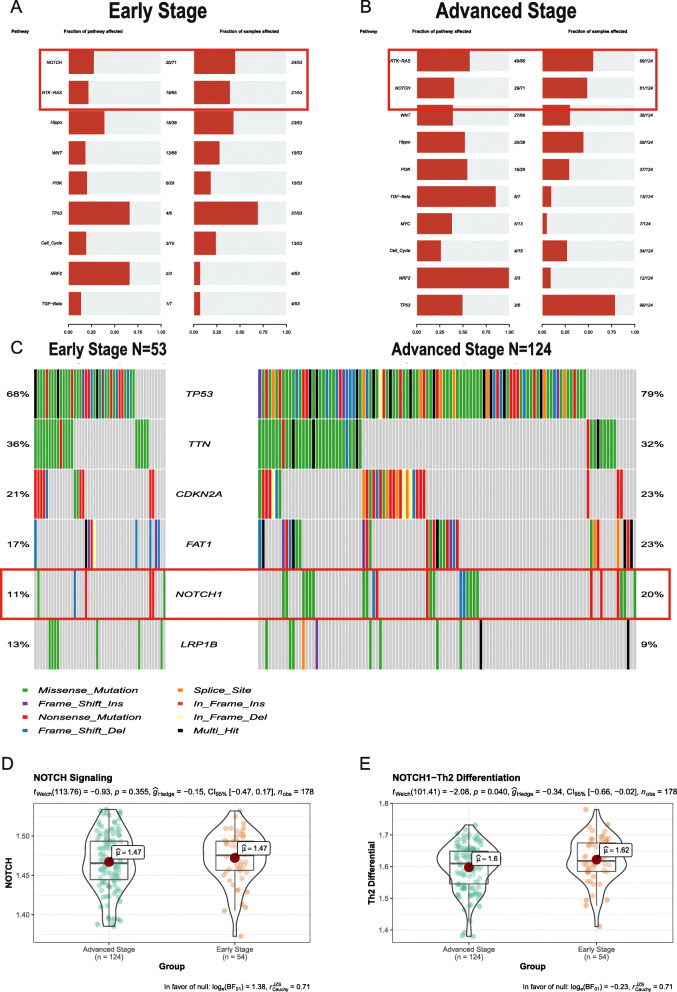
*NOTCH1* mutations in T/MF SCC samples decrease Th2 cell differentiation. Pathways enriched by mutated genes in (**A**) early- and (**B**) advanced-stage T/MF SCC samples. RTK-RAS and NOTCH signaling were the top two pathways in T/MF SCC. (**C**) Comparison of mutated genes between early- and advanced-stage T/MF SCC samples. *NOTCH1* mutations occurred significantly less in early-stage than in advanced-stage T/MF SCC (11% vs 20%). (**D**) NOTCH signaling activation score in early- and advanced-stage T/MF SCC calculated using ssGSEA. There was no statistically significant difference between the two groups. (**E**) NOTCH1-Th2 cell differentiation pathway activation score in early- and advanced-stage T/MF SCC calculated using ssGSEA. The NOTCH1-Th2 cell differentiation pathway was activated to a greater extent in early-stage than in advanced-stage T/MF SCC (*P* = 0.040)

To identify somatic mutations in patients with T/MF SCC at different stages, mutation data were downloaded and visualized using the Maftools package [12]. Enrichment analysis of all mutated genes in patients with early- and advanced-stage T/MF SCC indicated that the RTK-RAS and NOTCH1 pathways were the major function pathways affecting the development of T/MF SCC (Fig. 6A and B). Based on the waterfall plot, which shows the top six mutated genes in patients with T/MF SCC, it was inferred that *NOTCH1* mutations occurred significantly less in patients with early-stage T/MF SCC than in those with advanced-stage T/MF SCC (Fig. 6C). We then performed a ssGSEA to evaluate the activation level of the NOTCH1 signaling pathway. Gene signatures are shown in Table S1. The activation of the NOTCH1 signaling pathway did not change between patients with early- and advanced-stage disease (Fig. 6D). Studies have suggested that NOTCH1 can induce Th2 cell differentiation and regulate IL4 expression in Th2 cells [18, 19]. Therefore, data on gene signatures were derived from the KEGG analysis (Table S1), and we performed ssGSEA subsequently to evaluate the activation of the NOTCH1-Th2 cell differentiation pathway; results showed that in advanced-stage T/MF SCC, expression of this pathway was downregulated (Fig. 6E). This finding suggested that the reduction in Th2 cell numbers in advanced-stage T/MF SCC samples might be caused by the decrease in CCL22 expression and Th2 cell differentiation, which could be induced by mutated *NOTCH1*.

### CCL22 expression is positively correlated with GATA-3 and IL4 expression in tongue SCC samples

**Fig. 7:**
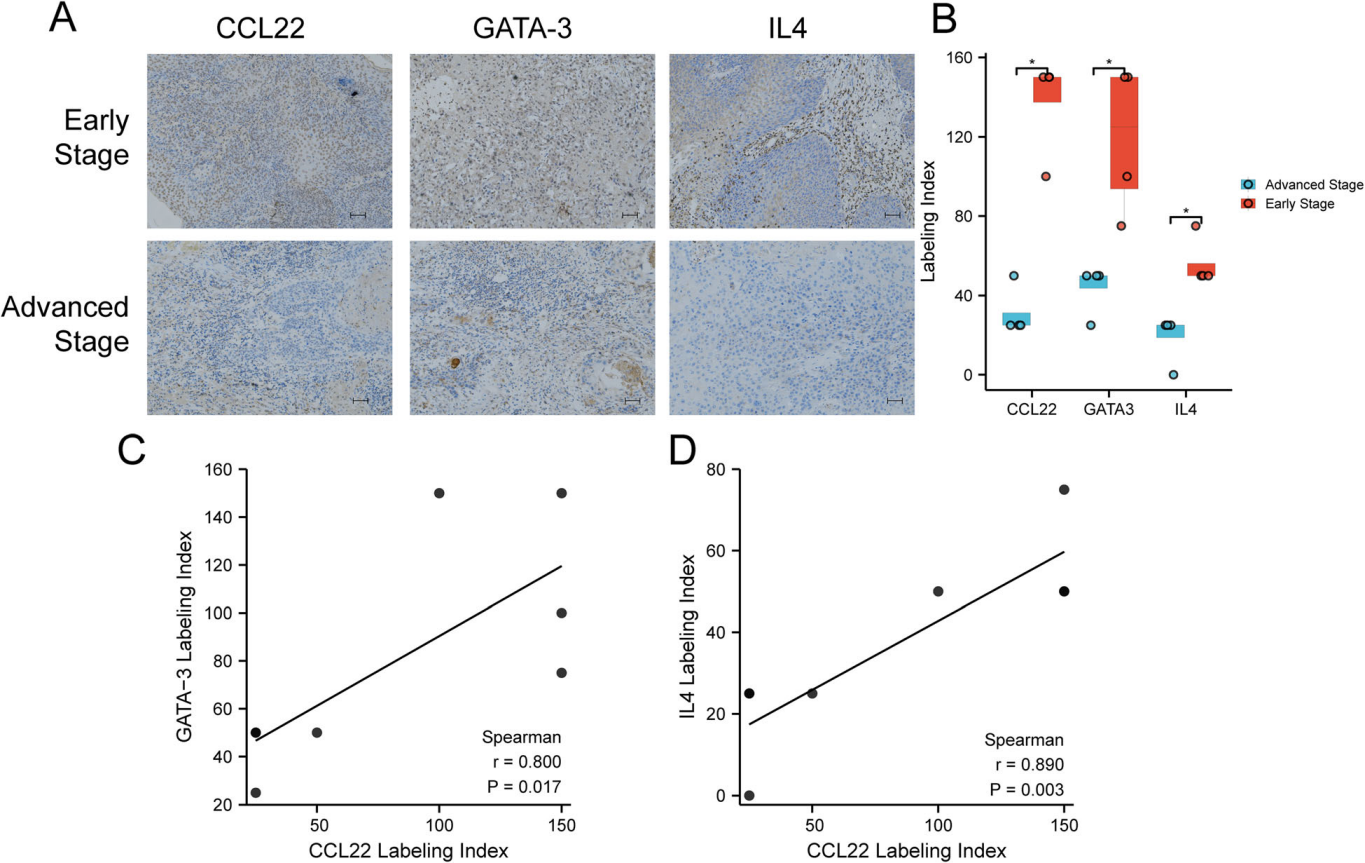
Protein levels of CCL22, GATA-3, and IL4 in tongue SCC tissues. (**A**) Immunohistochemical analysis of CCL22, GATA-3, and IL4 expression in tumor specimens between different clinical stages (× 200; scale bars = 50 μm). (**B**) Labeling index of CCL22, GATA-3, and IL4 in early- and advanced-stage T/MF SCC. The labeling index of CCL22 was positively correlated with that of (**C**) GATA-3 and (**D**) IL4. *P < 0.05

To verify the previously reported results, we performed immunohistochemical staining using eight paraffin-embedded tumor tissue samples to investigate the expression of CCL22, GATA-3, and IL4. Accordingly, CCL22, GATA-3, and IL4 were observed to be highly expressed in patients with early-stage tumors (Fig. 7A and B). Additionally, the expression of CCL22 was positively correlated with that of GATA-3 and IL4 in tongue SCC tissues (Fig. 7C and D).

## Discussion

The progression of T/MF SCC is affected by several factors, including gene mutation, dysregulation of long non-coding RNA, and changes in the TME [20–22]. With the recent development of single-cell sequencing technology, an increasing number of immune cell signatures have been generated, and the classification of immune cells in the TME has gained specificity [14, 23, 24]. Presently, the composition of the TME can be analyzed using RNA sequencing expression profiles.

Various mechanisms leading to the dysfunction of immune cells in the TME have been successively discovered. The immune microenvironment can directly or indirectly affect the occurrence and development of tumors. Its mechanisms include promotion of tumor angiogenesis, alteration of the biological characteristics of tumors cells, and establishment of an appropriate TME to promote tumor progression [4]. Liu et al. [25] suggested that an altered Treg/Th17 balance in tongue cancer might promote disease progression. Macrophages in the TME of tongue cancer promote tumor cell invasion and migration [26]. In the present study, Th2 cell numbers were found to be reduced in advanced-stage T/MF SCC compared to early-stage T/MF SCC samples. Th2 cells undergo differentiation from naïve CD4^+^ T cells and mainly mediate humoral immunity. The role of Th2 cells in tumors is unclear. Accumulating evidence suggests that increased Th2 cell numbers or cytokine levels secreted by Th2 cells indicate poor prognosis in colorectal, esophageal, and prostate cancer [9, 10, 27]. However, a study conducted on B cell NHL suggests that high expression of IL4, which is secreted by Th2 cells, is strongly correlated with reduced cancer proliferation and increased survival [11]. High Th2 cell counts and low Th17 cell counts were shown to be good prognosis factors in NSCLC [28]. Our results suggest that high Th2 cell numbers may play a protective role in T/MF SCC.

Changes in Th2 cell populations are related to the recruitment of Th2 cells and their differentiation level. CCL22 and CCL17 are chemokines that regulate Treg and Th2 cell recruitment via binding to their receptor CCR4. We found that there was no difference in CCL17 expression between early- and advanced-stage T/MF SCC samples, while CCL22 expression was significantly decreased in the latter compared to early-stage T/MF SCC samples. Overexpression of CCL22 in human tumors was reportedly associated with increased infiltration of Tregs, along with augmented tumor growth and poor prognosis in breast, gastric, and liver cancer [29–33]. Hirata et al. [34] suggested that CCL22 produced by naïve CD4^+^ T cells contributed to an increase in Th2 cell numbers in atopic diseases. However, studies conducted on CCL22 expression and Th2 cells in cancers are limited and indicate that CCL22 expression is a risk prognostic factor in breast and colorectal cancer [35, 36]. A study conducted on tongue cancer suggests that high expression of CCL22 influences the balance of M1- and M2-like macrophages and leads to a deteriorated prognosis [37]. Conversely, in the present study, CCL22 expression was found to be a protective prognostic factor in T/MF SCC. We quantified Tregs in patients with early- and advanced-stage T/MF SCC and found no statistically significant difference between both (Fig. S5). We speculate that CCL22 expression only affects the recruitment of Th2 cells, rather than Tregs, in patients with T/MF SCC, suggesting that CCL22 expression may affect the prognosis of these patients and the progression of T/MF SCC through the regulation of Th2 cells. This finding opens new avenues for the development of therapeutic approaches for T/MF SCC.

Gene mutation plays a key role in the development and progression of cancer. Recent large-scale genome sequencing efforts have validated *TP53* as the most commonly mutated gene in HNSCC [38]. Determination of the mutation landscape in tongue cancer revealed that *TP53*, *FAT1*, *CDKN2A*, *NOTCH1*, and *PIK3CA* were the most frequently mutated genes [39]. Herein, mutated *NOTCH1* expression was found at a significantly reduced level in patients with early-stage disease compared to those with advanced-stage disease. NOTCH signaling is involved in different types of malignant tumors [40] and has mostly been found to be altered in HNSCC [41]. Nevertheless, NOTCH can act as an oncogene or tumor suppressor depending on the cellular context [42]. The role of NOTCH in OSCC as a tumor suppressor has been previously suggested [38, 43, 44]. Activated NOTCH1 demonstrates an anti-proliferative function in tongue tumor cells through the downregulation of Wnt/β-catenin signaling, thereby inducing apoptosis and cell cycle arrest [45]. However, in our study, the activation level of the NOTCH1 signaling pathway did not change between both groups of patients. Evidence suggests that NOTCH1 can induce Th2 cell differentiation [18, 19]. We found that the activation level of the NOTCH-Th2 cell differentiation pathway was upregulated in patients with early-stage T/MF SCC. These results indicate that mutated *NOTCH1* may affect the activation of the NOTCH-Th2 cell differentiation pathway, thus reducing the number of Th2 cells.

Our study shows that increased expression of CCL22 in T/MF SCC may activate the recruitment of Th2 cells, while mutations in *NOTCH1* may inhibit Th2 cell differentiation. These two mechanisms influence the infiltration of Th2 cells in T/MF SCC, leading to disease progression. However, these conclusions were deduced only from results obtained via bioinformatics analysis; thus, our findings warrant further experimental verification. Additionally, the positions, identities, and effects of mutations in *NOTCH* genes can be cancer-specific and may reflect varied roles for NOTCH in different cancers [46]. Whether mutated *NOTCH* expression influences Th2 cell differentiation in T/MF SCC remains unclear and should be further explored.

In conclusion, in T/MF SCC, high expression of CCL22 promotes the recruitment of Th2 cells and helps predict better survival; moreover, mutations in *NOTCH1* inhibit the differentiation of Th2 cells. The decrease in Th2 cell recruitment and differentiation leads to tumor progression.

## Supplementary Information

**Additional file 1: **Supplementary Figure.

**Additional file 2: Table S1.** Gene signatures of NOTCH signaling and NOTCH1-Th2 differetiation Pathway.

## Data Availability

The data was downloaded from HNSCC projects of TCGA database (https://xenabrowser.net/datapages/?cohort=GDC%20TCGA%20Head%20and%20Neck%20Cancer%20(HNSC)&removeHub = https%3A%2F%2Fxena.treehouse.gi.ucsc.edu%3A443). This study meets the publication guidelines of TCGA. All data used in this study were obtained from TCGA for free.
