# Supplementary material for: Downregulation of CCL22 and mutated NOTCH1 in tongue and mouth floor squamous cell carcinoma results in decreased Th2 cell recruitment and expression, predicting poor clinical outcome
Source: BMC Cancer. 2021 Aug 15;21:922. doi: 10.1186/s12885-021-08671-1 (PMC8364714; doi:10.1186/s12885-021-08671-1)
Supplement: Supplementary file 1 — Additional file 1: Supplementary Figure. [file 12885_2021_8671_MOESM1_ESM.docx]

Supplementary Figure legends


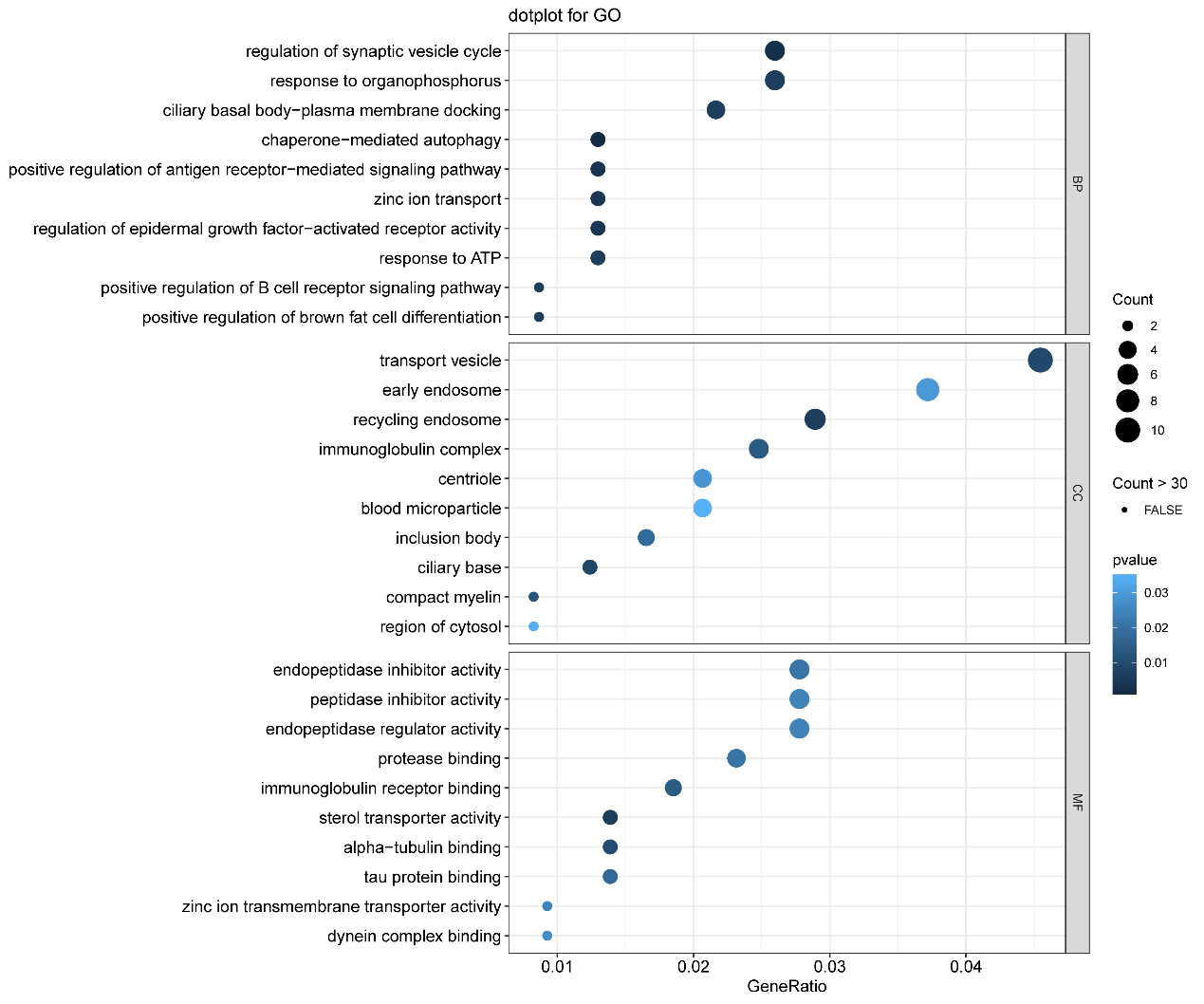


Figure.S1 The top 10 GO enrichment items in biological process (BP), molecular function (MF) and cellular component (CC) group of DEGs


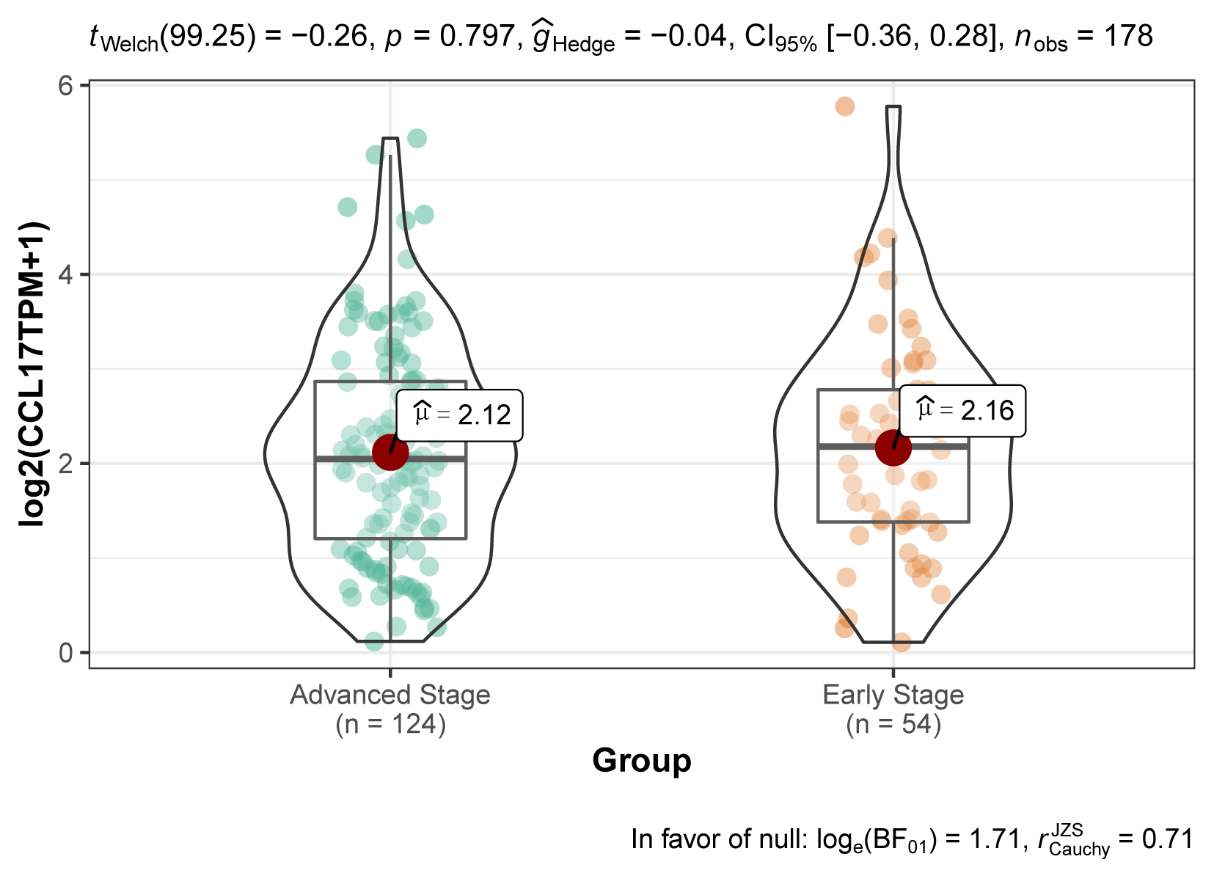


Figure.S2 The expression of CCL17 between Early Stage and Advanced Stage patients.


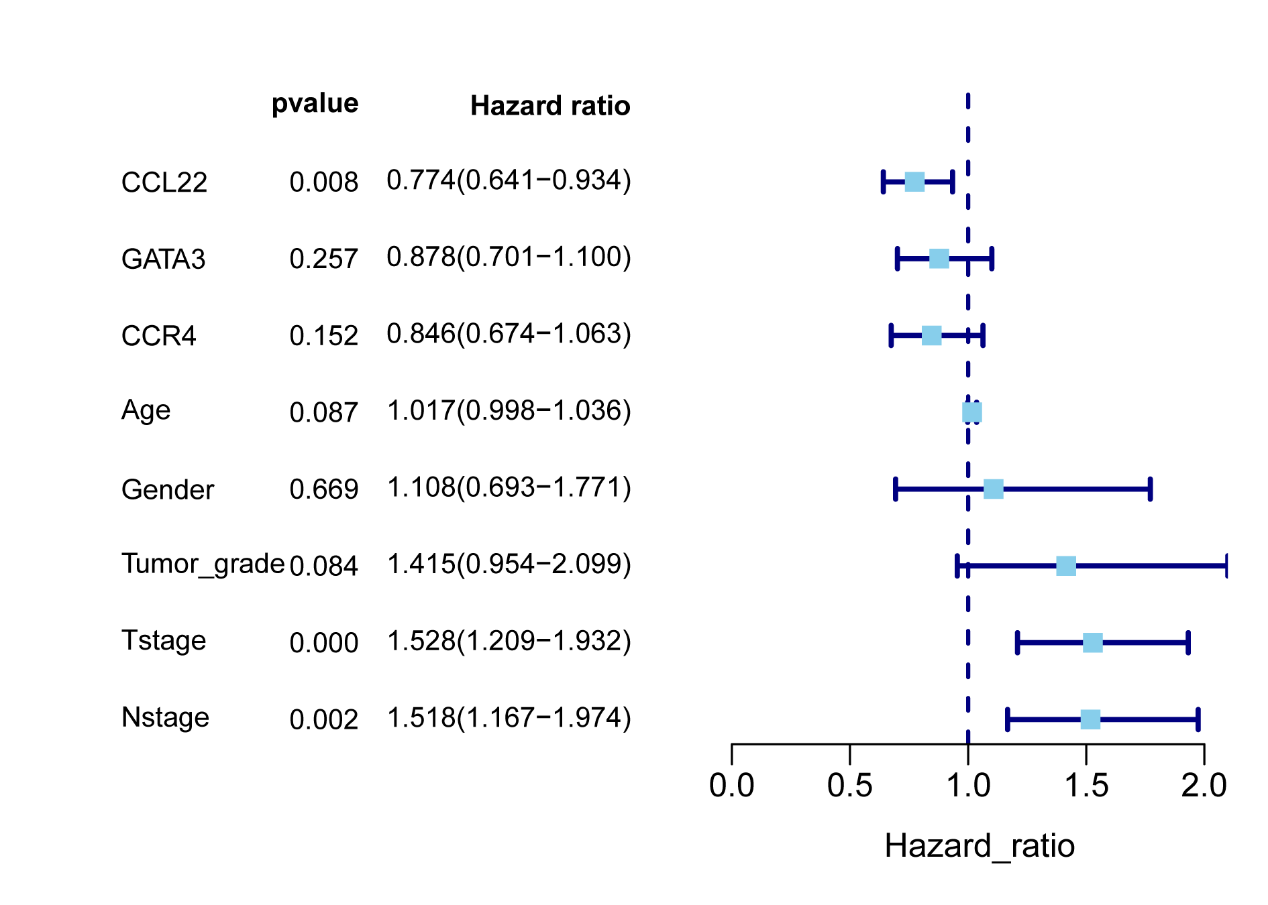


Figure.S3 Forest plot of univariate cox regression results.


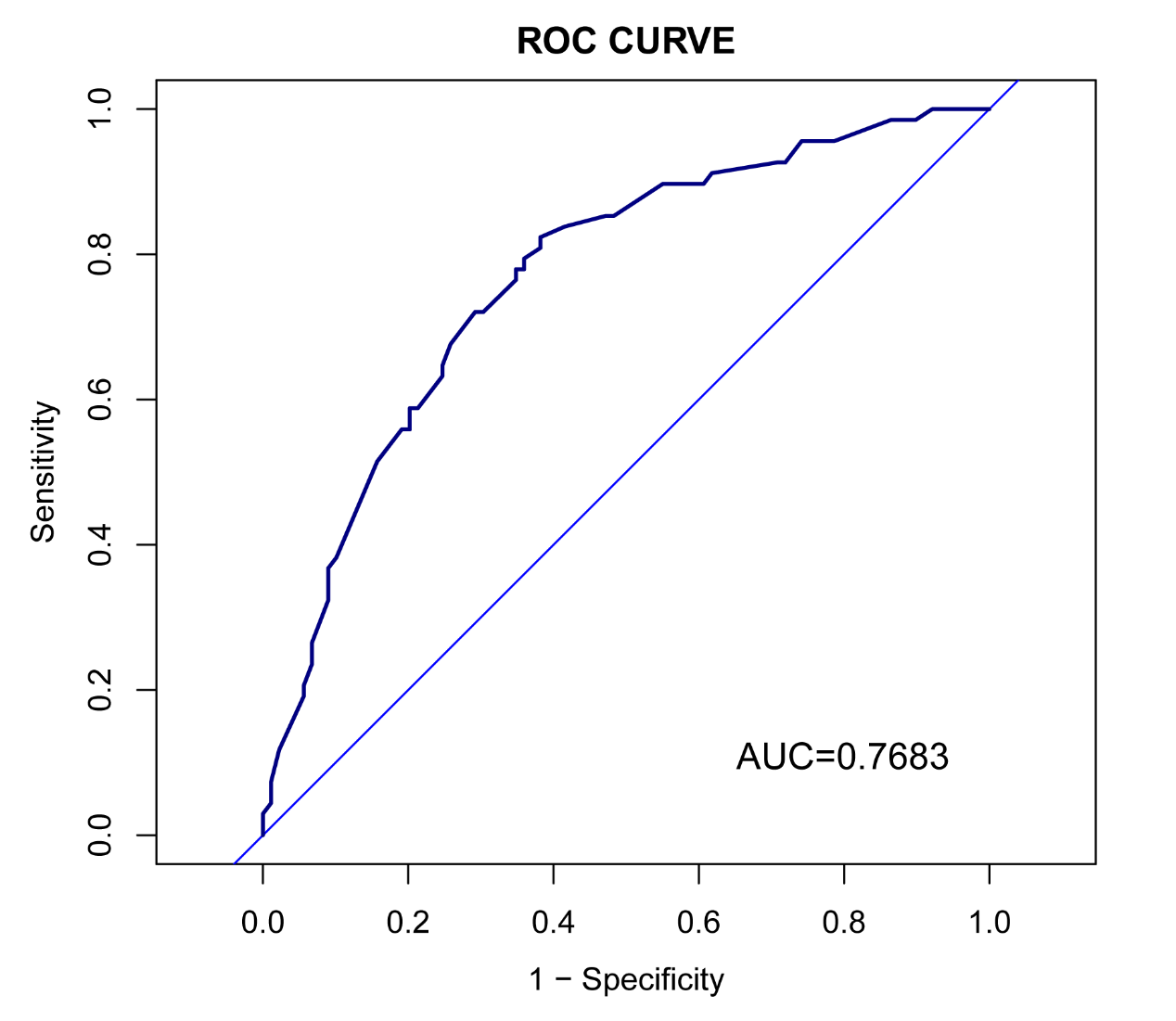


Figure.S4 The ROC of multivariate cox regression model.


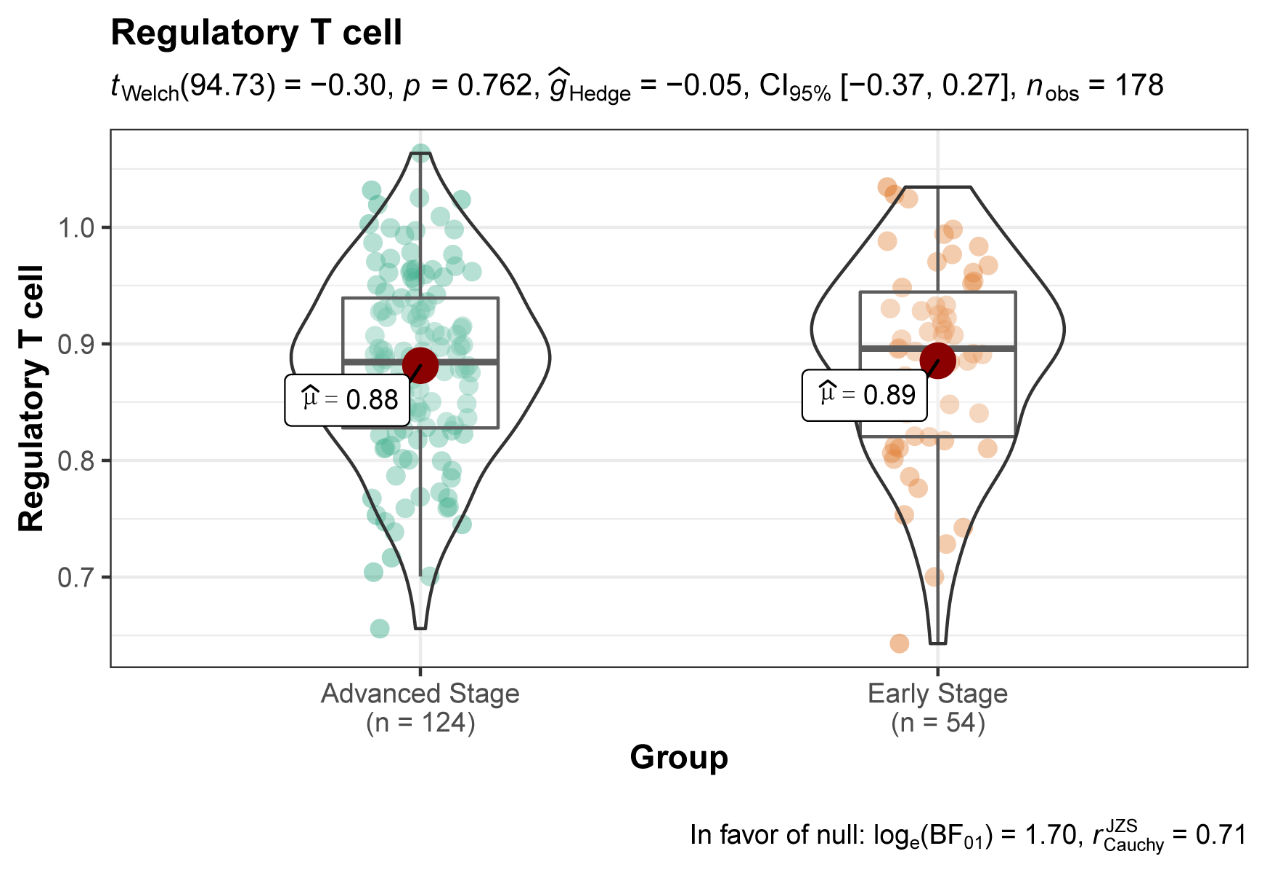


Figure.S5 The expression level of Treg cells between Early Stage and Advanced Stage patients using *28 Immune cells* signature.
