# Supplementary material for: Downregulation of CCL22 and mutated NOTCH1 in tongue and mouth floor squamous cell carcinoma results in decreased Th2 cell recruitment and expression, predicting poor clinical outcome
Source: BMC Cancer. 2021 Aug 15;21:922. doi: 10.1186/s12885-021-08671-1 (PMC8364714; doi:10.1186/s12885-021-08671-1)
Supplement: Supplementary file 2 — Additional file 2: Table S1. Gene signatures of NOTCH signaling and NOTCH1-Th2 differetiation Pathway. [file 12885_2021_8671_MOESM2_ESM.docx]

Table. S1. Gene signatures of NOTCH signaling and NOTCH1-Th2 differetiation Pathway

| Pathway | Genes |
| --- | --- |
| NOTCH Signaling | EGFL7,DTX3L,DTX3,DTX2,DLL4,DLL3,DLK1,APH1B,ADAM10,CTBP2,CTBP1,SNW1,SKP1,SAP30,RBX1,RBPJL,PSENEN,NUMBL,MAML2,MAML1,CNTN1,CIR1,MFNG,JAG2,NUMB,HDAC1,MFAP5,NCOR2,CUL1,MFAP2,HDAC2,THBS2,FHL1,NCSTN,ITCH,LFNG,PSEN2,NRARP,NOV,NOTCH4,NOTCH3,KAT2B,HEYL,HEY2,HEY1,HES5,HES4,HES3,HES2,HES1,ARRDC1,RFNG,PSEN1,NCOR1,DTX4,DTX1,DNER,DLL1,APH1A,ADAM17,SPEN,RBPJ,MAML3,KDM5A,JAG1,EP300,CNTN6,NOTCH2,CREBBP,FBXW7,NOTCH1 |
| NOTCH1-Th2 Differentiation | DLL,NOTCH3,MAML,RBPSUH,NFKB1,RELA,INFG,IFNGR1,IFNGR2,JAK1,JAK2,STAT1,IL12A,IL12B,IL12R,IL12RB1,IL12RB2,TYK2,STAT4,TBX21,RUNX3,MHC2,CD4,LCK,CD3E,CD3G,TRAV,TRBV,CD247,CD3D,LAT,ZAP70,PLCG1,PPP3C,PPP3R,NFATC2,NFATC3,IKBKA,PRKCQ,IKBKB,IKBKG,NFKBIA,NFKBIB,NFKBIE,ERK,FOS,P38,JNK,JUN,IL2,IL2RA,IL2RB,IL2RG,JAK3,STAT5A,STAT5B,IL4,IL4R,STAT6,GATA3,IL5,IL13,JAG1,JAG2,NOTCH1,NOTCH2,CMAF |

,,,,
